# Primary rectal squamous cell carcinoma resembling a submucosal tumor

**DOI:** 10.1002/jgh3.12514

**Published:** 2021-02-26

**Authors:** Yasuhiko Hamada, Kyosuke Tanaka, Youichirou Baba, Noriyuki Horiki

**Affiliations:** ^1^ Department of Gastroenterology and Hepatology Mie University Hospital Tsu Japan; ^2^ Department of Endoscopy Mie University Hospital Tsu Japan; ^3^ Department of Pathology Suzuka Central General Hospital Suzuka Japan

**Keywords:** clinical practice and treatment, colorectal cancer, endoscopy, gastroenterology, gastrointestinal oncology

## Abstract

Primary rectal squamous cell carcinoma is an extremely rare tumor and, in most cases, detected at an advanced stage. In our case, the tumor was at an early stage and had a submucosal appearance. Thus, the tumor was difficult to differentiate from other rectal submucosal tumors and, it was removed by endoscopic submucosal dissection for excisional biopsy.

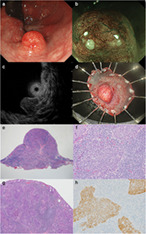

A 71‐year‐old woman presented with a 2‐month history of anal discomfort. Her past medical history included hyperlipidemia and retinitis pigmentosa. A colonoscopy revealed a submucosal tumor, 9 mm in size, in the lower rectum (Fig. 1a). Magnifying endoscopy with narrow‐band imaging revealed dilated vessels (Fig. 1b). Endoscopic ultrasonography detected a hypoechoic mass, which was confined to the submucosa (Fig. 1c). Although a carcinoid tumor was suspected, and the tumor was biopsied with conventional endoscopic forceps, the biopsy did not yield a definitive diagnosis. After discussion with the patient, she underwent an endoscopic submucosal dissection for excisional biopsy, without rebiopsy using endoscopic ultrasound fine‐needle biopsy or tunnel biopsy (Fig. 1d). Notably, the resected specimen revealed a squamous cell carcinoma (SCC) (Fig. 1e,f) with lymphatic involvement, which was covered with normal rectal epithelium (Fig. 1g). Immunohistochemical findings revealed the tumor cells to be positive for p16, suggesting coexisting human papilloma virus (HPV) infection (Fig. 1h). Metastasis from SCC‐related malignancy in another organ to the rectum was excluded because ^18^F‐fluorodeoxyglucose (FDG) positron emission tomography/computed tomography did not demonstrate any FDG‐avid area to suggest other malignancies, such as gynecologic, head and neck, esophageal, or lung carcinomas. In addition, continuity between the tumor and the normal anal squamous epithelium was not seen. Based on these findings, a diagnosis of primary rectal SCC was finally established. After discussion with the patient, she underwent additional chemoradiation therapy.

**Figure 1:**
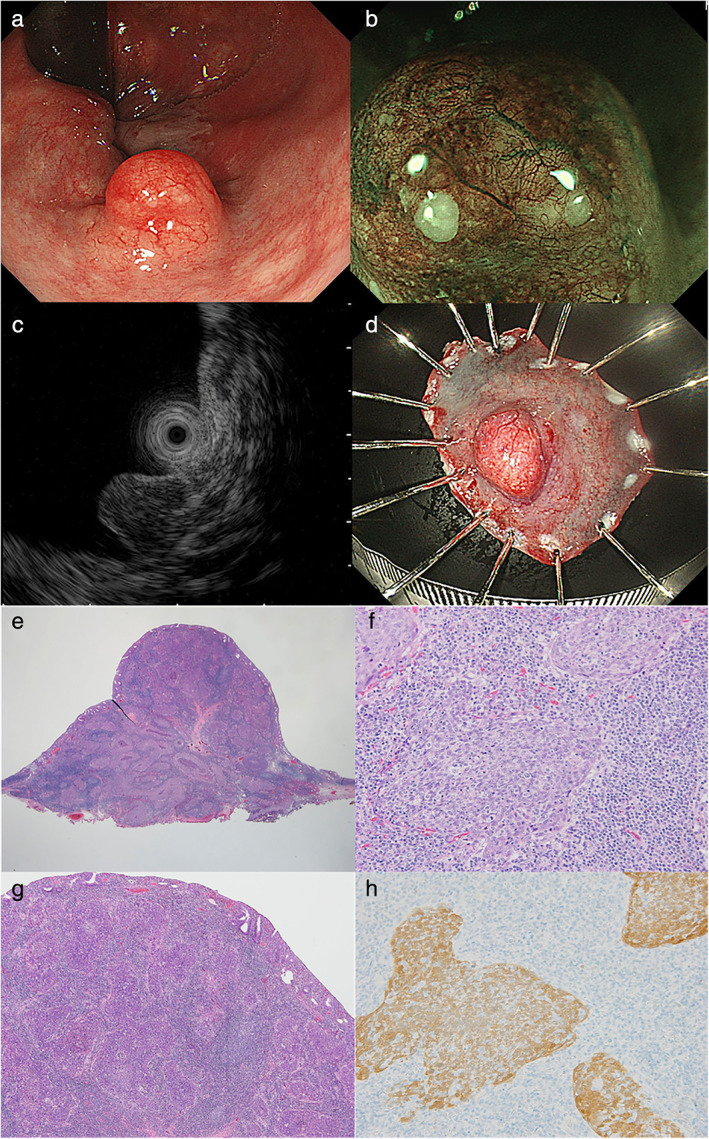
Colonoscopy revealed a 9 mm‐sized submucosal tumor (SMT) in the lower rectum (a). Magnifying endoscopy with narrow‐band imaging revealed dilated vessels (b). Endoscopic ultrasonography detected a hypoechoic mass, which was confined to the submucosa (c). The SMT was performed using en bloc resection by endoscopic submucosal dissection (d). Resected specimen revealed a squamous cell carcinoma (SCC) (e, ×12.5; f, ×200), which was covered with normal rectal epithelium (g, ×40). Immunohistochemical findings revealed the tumor cells to be positive for p16, suggesting coexisting human papilloma virus infection (h, ×200).

Primary rectal SCC is extremely rare and accounts for approximately 0.01–0.025% of all colorectal carcinomas. The pathogenesis remains under debate, yet metaplasia seems to be significantly responsible for transforming columnar to squamous epithelium. Given limited evidence, HPV infection may play a role in the pathogenesis of rectal SCC. The diagnosis of primary rectal SCC requires that three criteria are met: (i) exclusion of metastasis from other organs, (ii) no squamous‐lined fistulous tract involving the affected rectum, and (iii) exclusion of anal SCC with proximal extension (absence of continuity between the tumor and the normal anal squamous epithelium). Our patient fulfilled all these criteria. Although the endoscopic findings are poorly documented, some studies have reported that the tumor takes on a submucosal appearance, as in our case. This growth pattern can imply the proliferation of mucosal basal cells into squamous cells, which subsequently undergo malignant change. As such, primary rectal SCC should be considered a differential diagnosis in patients with rectal submucosal tumor.

